# *YTHDF3* gene polymorphisms increase Wilms tumor risk in Chinese girls

**DOI:** 10.7150/jca.99928

**Published:** 2024-10-07

**Authors:** Changmi Deng, Yufeng Han, Haixia Zhou, Jiao Zhang, Jiwen Cheng, Suhong Li, Jichen Ruan, Guochang Liu, Jing He, Rui-Xi Hua, Wen Fu

**Affiliations:** 1 Department of Pediatric Surgery, Guangzhou Institute of Pediatrics, Guangdong Provincial Key Laboratory of Research in Structural Birth Defect Disease, Guangzhou Women and Children's Medical Center, Guangzhou Medical University, Guangzhou 510623, Guangdong, China.; 2 Department of Hematology, The Key Laboratory of Pediatric Hematology and Oncology Diseases of Wenzhou, The Second Affiliated Hospital and Yuying Children's Hospital of Wenzhou Medical University, Wenzhou 325027, Zhejiang, China.; 3 Department of Pediatric Surgery, The First Affiliated Hospital of Zhengzhou University, Zhengzhou 450052, Henan, China.; 4 Department of Pediatric Surgery, The Second Affiliated Hospital of Xi'an Jiaotong University, Xi'an 710004, Shaanxi, China.; 5 Department of Pathology, Children Hospital and Women Health Center of Shanxi, Taiyuan 030013, Shannxi, China.

**Keywords:** *YTHDF3*, Wilms tumor, m^6^A, polymorphism, susceptibility

## Abstract

Wilms tumor is a prevalent pediatric tumor influenced by various genetic factors. m^6^A modification is a common nucleotide modification that plays a role in a variety of cancers. As a “reader”, YTHDF3 is essential for recognizing m^6^A modifications. However, the association between *YTHDF3* gene polymorphisms and Wilms tumor susceptibility has not been previously reported. A five-center case‒control study including 414 patients and 1199 controls was conducted to explore the relationship between *YTHDF3* gene polymorphisms and Wilms tumor susceptibility. The samples were genotyped via TaqMan real-time quantitative polymerase chain reaction. Odds ratios (ORs) and 95% confidence intervals (CIs) were utilized as indicators to assess their correlation. The *YTHDF3* rs2241753 AA genotype was significantly associated with an increased risk of Wilms tumor in females (adjusted OR=1.74, 95% CI=1.05-2.88, *P*=0.033). The risk of Wilms tumor was also notably elevated in female children with 1-3 risk genotypes (adjusted OR=1.47, 95% CI=1.04-2.07, *P*=0.028). The *YTHDF3* rs2241753 AA genotype and the presence of 1-3 risk genotypes were significantly associated with increased Wilms tumor risk in female children.

## Introduction

Wilms tumors, also known as nephroblastoma, are common childhood renal malignancies, accounting for 6% of pediatric oncologic diseases and 95% of renal tumors in children 1. The majority of cases occur in children under 5 years of age, with the highest incidence in children 2-3 years of age 1, 2. In Western countries, the incidence of Wilms tumor is approximately 7-10 cases per million people, whereas in China, it is approximately 3.3 cases per million people 3, 4. Wilms tumor manifests as an abnormality in the developmental process of the renal embryo, resulting in the coexistence of different stages of renal development, including persistent blastema, tubular epithelial and mesenchymal components 5, 6. It exhibits diverse morphological structures, and its histopathological features are believed to be correlated with its prognosis 6. Advancements in modern medicine have led to considerable success in treating Wilms tumor, which has an overall survival rate exceeding 90% 7, 8. However, notably, patients with adverse histologic features still have a poor prognosis, and the survival rate for patients with recurrence remains relatively low 8. In addition, approximately 25% of survivors have serious chronic diseases 9. Therefore, further refinement in the treatment of Wilms tumor is necessary to reduce the incidence of complications and sequelae and to develop therapeutic approaches for high-risk Wilms tumor types.

Genetic factors are widely recognized to play crucial roles in the development of Wilms tumor. The inactivation of WT1, the first identified tumor suppressor gene associated with Wilms tumor, is an important factor in Wilms tumor development 10. Subsequent studies have revealed that mutations in *WTX*, *TP53*, *CTNNB1*, *IGF2*, and *MYCN* affect the development of Wilms tumor 11-13. There is increasing strong evidence supporting the contribution of genetic variation to Wilms tumor. Single nucleotide polymorphisms (SNPs) are common genetic variants that affect cancer susceptibility. A genome-wide association study (GWAS) revealed the presence of genes associated with Wilms tumor susceptibility in the 2p24 and 11q14 regions. Moreover, 22q12, Xp22 and 5q14 were also predicted to be potential risk regions 14. Studies on candidate genes have shown that polymorphisms in base excision repair genes and nucleotide excision repair genes are significantly associated with Wilms tumor susceptibility 5, 15. There is evidence demonstrating an association between Wilms tumor susceptibility and polymorphisms in various tumor-associated genes, such as *BARD1* 16, *METTL3* 17, *FTO* 18, and *ALKBH5* 19. However, further exploration into the genetic etiology of Wilms tumor is warranted.

N^6^-methyladenosine (m^6^A) is a common, widely distributed posttranscriptional modification of mRNAs in eukaryotes 20. Mounting evidence suggests that m^6^A modification plays a pivotal role in cancer development and regulation 21-23. This modification is regulated by "writers", "erasers", and "readers" 24, 25. RNA methyltransferases, known as "writers", primarily include METTL3/METTL14/WTAP, which are responsible for adding methyl groups to the adenine residues of mRNAs 26, 27. Demethylases such as FTO and ALKBH5 act as "erasers" by removing methylations. YTHDF1/2/3 and IGF2BP1 are m^6^A-binding proteins referred to as “readers”, which specifically recognize and bind to m^6^A modification sites on mRNAs and play essential roles in regulating mRNA stability, translation, splicing and export 28-31. Consequently, dysregulation of m^6^A modification is often closely associated with various diseases, especially cancer 32, 33.

YTHDF3, a member of the YTH domain family, is believed to regulate the translation and degradation of methylated mRNAs in concert with YTHDF1 and YTHDF2 29. Currently, the relationship between *YTHDF3* gene variants and cancers such as Wilms tumor remains unclear. The associations between SNPs of* YTHDF2* and *YTHDC1* (other members of the YTH domain family) and Wilms tumor susceptibility have been elucidated 34, 35. However, further analysis is needed to understand the relationship between the *YTHDF3* gene SNPs and the risk of Wilms tumor. The objective of this study was to investigate the association between *YTHDF3* polymorphisms and Wilms tumor susceptibility in Chinese children while providing a theoretical basis for subsequent studies.

## Materials and methods

### Study population

The participation criteria and clinical characteristics of the selected subjects have been described in previous studies 19. Patients with Wilms tumor who were included were required to meet the following criteria: (1) Han Chinese ancestry; (2) new diagnosis confirmed by pathology; (3) no family history of disease or cancer; and (4) age 14 years or younger. Patients who had received medical interventions or did not provide signed informed consent were excluded. Ultimately, we recruited 414 patients who were diagnosed with Wilms tumor from five cities in China and 1199 healthy controls who were matched for age and sex (**Table S1**). All the subjects were from the Chinese Han population. The parents or guardians of the subjects signed an informed consent form. The study was approved by the Ethics Committee of Guangzhou Women and Children's Medical Center (Ethical Approval No: 202016601).

### Polymorphism selection and genotyping

SNPs with potential functions in *YTHDF3* were screened via the dbSNP database (https://www.ncbi.nlm.nih.gov/snp), SNPinfo software (http://snpinfo.niehs.nih.gov/), and LDlink (https://ldlink.nci.nih.gov/), and the screening criteria were described in detail in previous studies 36. Briefly, the following criteria were used: (1) SNPs located at both ends of the *YTHDF3* gene, i.e., the 5' nearest gene, the 3' nearest gene, and the 3' and 5' untranslated regions (UTRs); (2) SNPs with a minor allele frequency (MAF) ≥ 5% in Chinese Han subjects; (3) SNPs affecting the activity of transcription factor-binding sites (TFBSs) or miRNA binding sites; and (4) SNPs with a low linkage disequilibrium (LD) with other selected SNPs (R^2^<0.8). Ultimately,* YTHDF3* rs2241753 G>A, rs2241754 A>G, and rs7464 A>G were selected for further study; rs2241753 G>A and rs2241754 A>G affect TFBS activity and rs7464 A>G affect miRNA binding site activity** (Table S2)**. There was no significant LD among these selected SNPs (R^2^<0.8). R^2^=0.372 between rs2241753 G>A and rs2241754 A>G; R^2^=0.178 between rs2241753 G>A and rs7464 A>G; R^2^=0.149 between rs2241754 G>A and rs7464 A>G (**Figure S1**).

Genomic DNA was extracted from peripheral blood via the TIANamp Blood DNA Kit (TianGen Biotech, Beijing, China). DNA samples were genotyped for SNPs via TaqMan real-time PCR (Applied Biosystems, Foster City, CA) 18, 37. To ensure accuracy, negative controls without DNA templates were included on each plate, and laboratory technicians were blinded to the sample information. Additionally, a random selection of 10% of the samples was regenerated to assess the error rate, with the results showing 100% concordance.

### Statistical analysis

Depending on the type of variable, either the chi-square test or t test was used to compare the clinical differences between the patients and controls. The degree of deviation of each SNP genotype from Hardy‒Weinberg equilibrium (HWE) in the controls was assessed via a goodness-of-fit chi-square test. The homozygotes of the common allele were designated as the reference group, whereas the remaining genotypes were classified as variants 38. An unconditional logistic regression analysis was conducted to examine the association between *YTHDF3* SNPs and Wilms tumor risk, with adjustments made for age and sex to minimize their potential interference with the correlation analysis. Odds ratios (ORs) and 95% confidence intervals (CIs) were employed as indicators to evaluate this correlation. The effects of the *YTHDF3* SNPs on Wilms tumor susceptibility were subsequently analyzed in patients stratified by age, sex, and clinical stage. eQTL analysis was performed via the GTEx portal (https://www.gtexportal.org/home/) to determine the effects of SNPs on nearby gene expression levels. All the statistical significance tests were performed with a two-sided *P*<0.05 as the threshold. All data analysis was performed via SAS 9.1 (SAS Institute Inc.).

## Results

### Association between *YTHDF3* polymorphisms and Wilms tumor risk

The *YTHDF3* SNPs were successfully genotyped in 391 of 414 cases and 1198 of 1199 controls. The correlation between *YTHDF3* gene polymorphisms and Wilms tumor susceptibility is shown in **Table 1**. The genotype frequencies of all three SNPs in the control group were consistent with HWE (HWE>0.05). However, none of the three SNPs were significantly correlated with Wilms tumor risk. Subsequently, rs2241753 AA, rs2241754 AA, and rs7464 AG/GG were identified as risk genotypes. However, combined analysis revealed no significant difference in Wilms tumor susceptibility between carriers of 1-3 risk genotypes and noncarriers.

### Subgroup analysis

Subgroup analysis was conducted by stratifying the subjects on the basis of age, sex, and clinical stage. The results are presented in **Table 2**. The rs2241753 AA genotype was significantly associated with an increased risk of Wilms tumor in the female subgroup (adjusted OR=1.74, 95% CI=1.05-2.88, *P*=0.033). Furthermore, the presence of 1-3 risk genotypes was also significantly associated with an increased risk of Wilms tumor in this subgroup (adjusted OR=1.47, 95% CI=1.04-2.07, *P*=0.028).

### Effect of rs2241753 G>A on the mRNA expression of nearby genes

**Figure 1:**
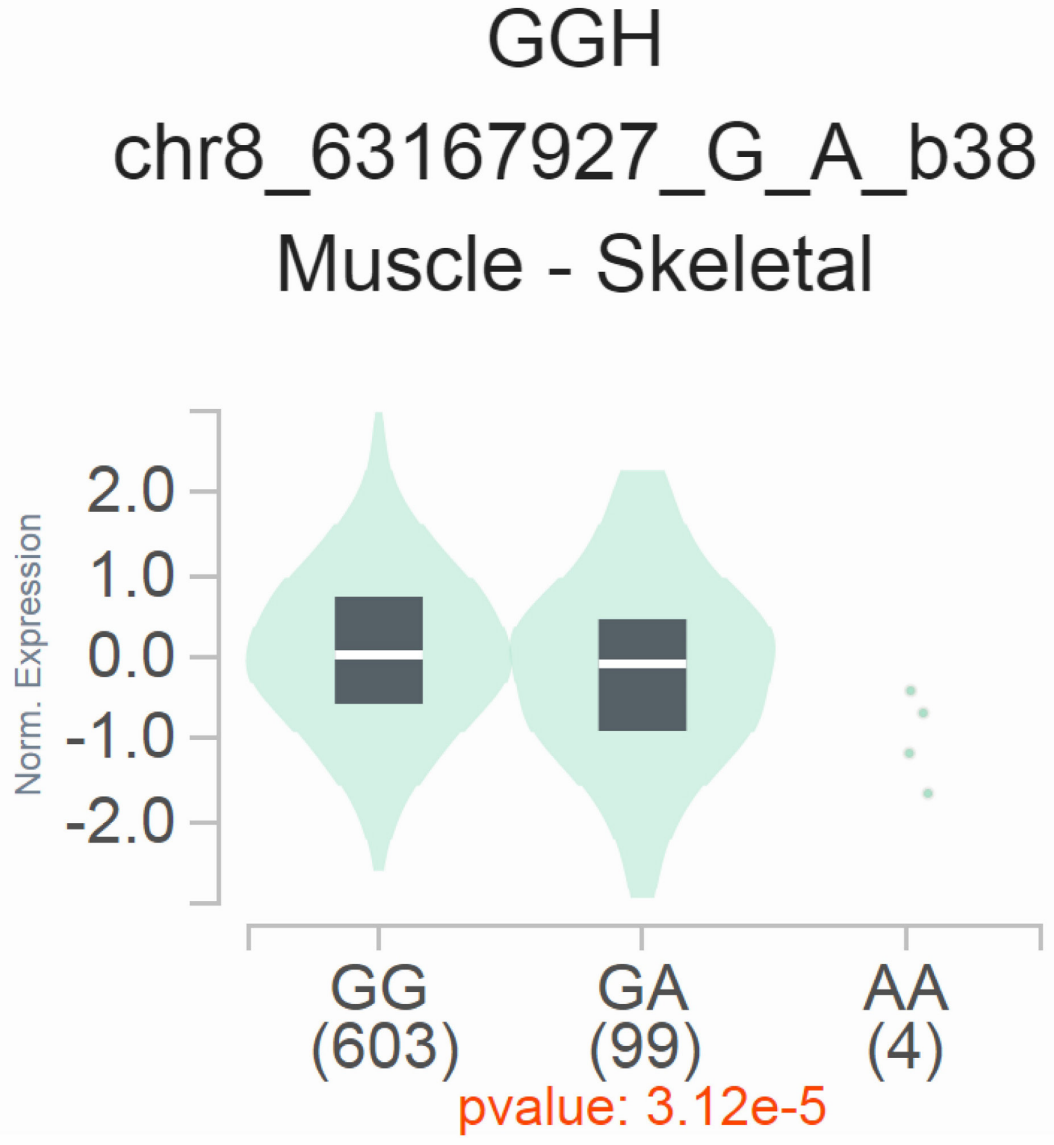
eQTL analysis of *YTHDF3* rs2241753 G>A in the GTEx database. The rs2241753 G genotype was significantly associated with increased *GGH* mRNA levels in skeletal muscle tissue (*P*=3.12×10^-5^).

We conducted expression quantitative trait locus (eQTL) analyses to explore the potential impact of rs2241753 G>A on the mRNA expression of its neighboring genes, utilizing data from GTEx. The results revealed a significant association between the presence of the rs2241753 G allele and increased *GGH* mRNA expression in skeletal muscle (**Figure 1**).

## Discussion

The m^6^A modification gene plays a crucial role in cancer regulation, and previous studies have partially elucidated the correlation between m^6^A gene SNPs and Wilms tumor susceptibility 17-19, 38. Here, we sought to investigate the potential role of *YTHDF3* gene SNPs in the risk of Wilms tumor. Our data indicate that rs2241753 in *YTHDF3* is significantly associated with an increased risk of Wilms tumor in female children. Furthermore, there is an increased risk of Wilms tumor in female children with 1-3 risk genotypes.

YTH domain family proteins, including YTHDF1, YTHDF2, YTHDF3, and YTHDC1, are essential for mediating m^6^A functions. YTHDC1 is responsible for regulating mRNA splicing and export in the nucleus 39. Unlike YTHDC1, YTHDF 1/2/3 are cytoplasmic m^6^A readers 40. YTHDF2 binds to m^6^A-modified mRNAs in the cytoplasm and localizes them to mRNA decay sites such as processing bodies, thereby promoting mRNA degradation 30. YTHDF1 interacts with initiation factors to promote ribosome production and ultimately enhances the translational efficiency of its target mRNAs 41. In addition, YTHDF3 potentially affects the specificity of YTHDF1 and YTHDF2 binding to mRNAs. YTHDF3 can act synergistically with YTHDF1 and YTHDF2 to affect the translation and degradation of m^6^A-modified mRNAs 29, 31. Therefore, YTHDF3 affects normal biological functions by regulating the translation and degradation of m^6^A-modified transcripts 29. Dysregulation of its expression may contribute to biological abnormalities and ultimately to diseases, including cancer.

YTHDF3 can synergize with poly(A)-binding protein cytoplasmic 1 (PABPC1) and eukaryotic translation initiation factor 4gamma2 (eIF4G2), but not YTHDF1, to bind to the m^6^A motif in the 5'UTR of *CCND1* mRNA and promote *CCND1* translation. CCND1 can further increase the renewal capacity of hematopoietic stem cells 42. In addition, YTHDF3 has been found to induce cellular autophagy in response to nutritional deficiencies. YTHDF3 expression is significantly increased in malnourished mouse embryonic fibroblasts (MEFs), and it promotes *FOXO3* translation by recognizing the m^6^A sites of *FOXO3* mRNA, which further induces cellular autophagy 43, 44. Furthermore, YTHDF3 is capable of negatively regulating antiviral immunity by upregulating FOXO3 to inhibit the expression of interferon-stimulated genes (ISGs) 45. Notably, during the induction of autophagy, YTHDF3 is believed to bind to m^6^A around the termination codon of the *FOXO3* transcript, promoting the translation of *FOXO3* by recruiting eukaryotic translation initiation factor 3 subunit A (eIF3a) and eukaryotic initiation factor 4B (eIF4B) at the 5' end 43. Conversely, in regulating antiviral immunity, YTHDF3 is believed to bind directly to the start region of the *FOXO3* mRNA in a non-m^6^A-dependent manner, promoting the translation of *FOXO3* by synergizing with PABP1 and eIF4G2 45. According to previous reports, the overexpression of *YTHDF3* induces brain metastasis in breast cancer patients, which leads to a poor patient prognosis. YTHDF3 promotes the translation of m^6^A-modified mRNAs of breast cancer brain metastasis-associated genes (e.g., *ST6GALNAC5*, *GJA1*, and *EGFR*) by recruiting eIF3a and increasing the expression of the corresponding proteins, which ultimately affects the brain metastasis of breast cancer 46. YTHDF3 also enhances its own translation efficiency by binding to the m^6^A residue of the 5'UTR of its mRNA. In addition, the copy number of the *YTHDF3* gene was found to be significantly increased in breast cancer, which led to the transcriptional upregulation of *YTHDF3* 46, 47. Patients harboring genetic alterations in *YTHDF3* have a relatively poor survival rate 47. Moreover, miR-106b-5p was found to downregulate *YTHDF3* expression in breast cancer 48. Both the mRNA and protein levels of YTHDF3 increased under the condition of glucose deficiency. *YTHDF3* overexpression was found to increase the expression of glycolytic genes and promotes the degradation of the antisense RNA DICER1 (DICER1-AS1). DICER1-AS1 restricts glycolysis, proliferation and metastasis in PC cells and acts as a PC inhibitory factor. The effect of YTHDF3 on DICER1-AS1 was found to be significantly enhanced in glucose-deficient pancreatic cancer cells. In addition, miR-5586-5p counteracts the inhibitory effect of YTHDF3 on DICER1-AS1 49.

On the basis of the aforementioned findings, *YTHDF3* overexpression promotes cancer. Differential analysis utilizing the TCGA database revealed significant differences in m^6^A modification regulatory genes between Wilms tumor tissues and normal tissues, with upregulation of *YTHDF3* expression in Wilms tumor tissues 50. The relationship between *YTHDF3* gene polymorphisms and Wilms tumor remains incompletely understood. Moreover, the effect of genetic variation in *YTHDF3* on its protein expression has not been adequately investigated, and whether *YTHDF3* SNPs affect its mRNA stability or protein expression levels needs to be further verified. Our study revealed that the *YTHDF3* rs2241753 AA genotype has an enhancing effect on Wilms tumor susceptibility in female children. The mechanism underlying this effect requires additional investigation, possibly because the variant of *YTHDF3* rs2241753 affects the expression of its downstream Wilms tumor-associated genes. *YTHDF3* rs2241753 is located in the TFBS, and single nucleotide variants here may affect the binding of mRNA to transcription factors, which in turn causes changes in gene expression levels. Furthermore, female carriers of 1-3 risk genotypes also exhibited increased Wilms tumor susceptibility, suggesting that a potential interaction among these variants impacts the risk of Wilms tumor. However, no significant association was observed between *YTHDF3* SNPs and Wilms tumor susceptibility in the overall sample included our study. This may be because the effect of *YTHDF3* SNPs on Wilms tumor is relatively subtle or population specific.

Our study presents a multicenter analysis with a large sample size, demonstrating for the first time the relationship between *YTHDF3* SNPs and Wilms tumor susceptibility. However, there are some limitations that should be acknowledged. First, the study was restricted to one race, as all participants were from the Chinese Han population. Second, the effects of lifestyle and environmental factors on Wilms tumor incidence were not considered. Third, the number of SNPs in the study was relatively small, indicating the need to include more *YTHDF3* SNPs and analyze their combined effects on Wilms tumor risk. Finally, additional protein-level studies are needed to determine the potential mechanisms by which *YTHDF3* SNPs influence Wilms tumor susceptibility.

In conclusion, our findings suggest that the presence of the *YTHDF3* rs2241753 AA genotype or 1-3 *YTHDF3* risk genotypes may increase the risk of Wilms tumor occurrence in female children. Our findings need to be validated in different populations after controlling for multiple confounders.

## Supplementary Material

Supplementary figure and tables.

## Figures and Tables

**Table 1:** Association of *YTHDF3* gene polymorphisms with Wilms tumor susceptibility

Genotype	Cases (N=391)	Controls (N=1198)	*P* ^ a^	Crude OR (95% CI)	*P*	Adjusted OR (95% CI) ^b^	*P* ^ b^
rs2241753 G>A (HWE=0.273)							
GG	182 (46.55)	538 (44.91)		1.00		1.00	
GA	163 (41.69)	542 (45.24)		0.89 (0.70-1.13)	0.342	0.89 (0.70-1.13)	0.341
AA	46 (11.76)	118 (9.85)		1.15 (0.79-1.69)	0.464	1.15 (0.78-1.68)	0.482
Additive			0.943	1.01 (0.85-1.20)	0.943	1.00 (0.85-1.20)	0.960
Dominant	209 (53.45)	660 (55.09)	0.572	0.94 (0.74-1.18)	0.572	0.94 (0.74-1.18)	0.565
GG/GA	345 (88.24)	1080 (90.15)		1.00		1.00	
AA	46 (11.76)	118 (9.85)	0.280	1.22 (0.85-1.75)	0.280	1.21 (0.85-1.74)	0.294
rs2241754 A>G (HWE=0.672)							
AA	125 (31.97)	380 (31.72)		1.00		1.00	
AG	190 (48.59)	583 (48.66)		0.99 (0.76-1.29)	0.944	1.00 (0.77-1.30)	0.996
GG	76 (19.44)	235 (19.62)		0.98 (0.71-1.37)	0.919	0.98 (0.71-1.36)	0.914
Additive			0.917	0.99 (0.84-1.17)	0.917	0.99 (0.84-1.17)	0.924
Dominant	266 (68.03)	818 (68.28)	0.927	0.99 (0.77-1.26)	0.927	1.00 (0.78-1.27)	0.970
AA/AG	315 (80.56)	963 (80.38)		1.00		1.00	
GG	76 (19.44)	235 (19.62)	0.938	0.99 (0.74-1.32)	0.939	0.98 (0.74-1.31)	0.899
rs7464 A>G (HWE=0.728)							
AA	207 (52.94)	662 (55.26)		1.00		1.00	
AG	152 (38.87)	454 (37.90)		1.07 (0.84-1.36)	0.579	1.08 (0.85-1.37)	0.554
GG	32 (8.18)	82 (6.84)		1.25 (0.81-1.93)	0.321	1.26 (0.81-1.95)	0.301
Additive			0.317	1.10 (0.92-1.31)	0.317	1.10 (0.92-1.32)	0.294
Dominant	184 (47.06)	536 (44.74)	0.424	1.10 (0.87-1.38)	0.424	1.10 (0.88-1.39)	0.400
AA/AG	359 (91.82)	1116 (93.16)		1.00		1.00	
GG	32 (8.18)	82 (6.84)	0.373	1.21 (0.79-1.86)	0.373	1.22 (0.80-1.87)	0.356
Combined effect of risk genotypes ^c^							
0	163 (41.69)	541 (45.16)		1.00		1.00	
1-3	228 (58.31)	657 (54.84)	0.231	1.15 (0.91-1.45)	0.231	1.16 (0.92-1.46)	0.221

OR, odds ratio; CI, confidence interval, HWE, Hardy‒Weinberg equilibrium. ^a^ c^2^ test for genotype distributions between Wilms tumor patients and cancer-free controls. ^b^ Adjusted for age and gender. ^c^ Risk genotypes were carriers with rs2241753 AA, rs2241754 AA and rs7464 AG/GG genotypes.

**Table 2:** Stratification analysis for association between *YTHDF3* genotypes and Wilms tumor susceptibility

Variables	rs2241753 (case/control)		AOR (95% CI)^a^	*P* ^a^	rs7464 (case/control)		AOR (95% CI)^a^	*P* ^a^	Risk genotypes (case/control)		AOR (95% CI)^a^	*P* ^a^
	GG/GA	AA			AA/AG	GG			0	1-3		
Age, months												
≤18	119/416	18/49	1.32 (0.74-2.35)	0.352	125/436	12/29	1.37 (0.68-2.78)	0.382	61/210	76/255	1.02 (0.70-1.50)	0.907
>18	226/664	28/69	1.22 (0.76-1.94)	0.409	234/680	20/53	1.09 (0.63-1.86)	0.766	102/331	152/402	1.25 (0.94-1.67)	0.132
Sex												
Female	157/474	27/47	**1.74 (1.05-2.88)**	**0.033**	167/494	17/27	1.86 (0.99-3.51)	0.053	72/253	112/268	**1.47 (1.04-2.07)**	**0.028**
Male	188/606	19/71	0.86 (0.50-1.46)	0.574	192/622	15/55	0.88 (0.48-1.59)	0.661	91/288	116/389	0.94 (0.69-1.29)	0.712
Clinical stage												
I	109/1080	19/118	1.60 (0.94-2.70)	0.082	122/1116	6/82	0.69 (0.29-1.62)	0.392	49/541	79/657	1.33 (0.91-1.94)	0.136
II	97/1080	15/118	1.41 (0.79-2.50)	0.246	102/1116	10/82	1.36 (0.68-2.71)	0.383	54/541	58/657	0.89 (0.60-1.31)	0.557
III	81/1080	8/118	0.93 (0.44-1.96)	0.840	83/1116	6/82	1.00 (0.42-2.36)	0.993	35/541	54/657	1.27 (0.82-1.98)	0.284
IV	43/1080	2/118	0.43 (0.10-1.81)	0.250	39/1116	6/82	2.14 (0.88-5.23)	0.094	17/541	28/657	1.37 (0.74-2.54)	0.313
I+II	206/1080	34/118	1.50 (1.00-2.26)	0.053	224/1116	16/82	0.99 (0.57-1.73)	0.976	103/541	137/657	1.10 (0.83-1.45)	0.516
III+IV	124/1080	10/118	0.75 (0.38-1.48)	0.410	122/1116	12/82	1.37 (0.73-2.59)	0.332	52/541	82/657	1.31 (0.91-1.89)	0.152

AOR, adjusted odds ratio; CI, confidence interval. ^a^ Adjusted for age and sex without the stratify factor.

## Abbreviations

SNP: single nucleotide polymorphism
GWAS: genome-wide association study
m^6^A: N^6^-methyladenosine
UTR: untranslated region
TFBS: transcription factor-binding site
LD: linkage disequilibrium
HWE: Hardy‒Weinberg equilibrium
OR: odds ratio
CI: confidence interval
eQTL: expression quantitative trait loci
PABPC1: poly (A) binding protein cytoplasmic 1
eIF4G2: eukaryotic translation initiation factor 4 gamma 2
MEF: mouse embryonic fibroblast
ISG: interferon-stimulated gene
eIF3a: eukaryotic translation initiation factor 3 subunit A
eIF4B: eukaryotic initiation factor 4B
DICER1-AS1: antisense RNA1 of DICER1
