# Supplementary material for: YTHDF3 gene polymorphisms increase Wilms tumor risk in Chinese girls
Source: J Cancer. 2024 Oct 7;15(18):6103–9. doi: 10.7150/jca.99928 (PMC11493011; doi:10.7150/jca.99928)
Supplement: Supplementary file 1 — Supplementary figure and tables. [file jcav15p6103s1.pdf]

Table S1. Frequency distribution of selected variables in Wilms tumor patients and cancer-free controls

Variables	Cases (N=414)		Controls (N=1199)		P [a]
	No.	%	No.	%	
Age range, month	1.00-148.63		0.03-156		0.118
Mean ± SD	31.14 ± 24.27		32.31 ± 26.15		
≤18	143	34.54	466	38.87	
>18	271	65.46	733	61.13	
Sex					0.218
Female	194	46.86	520	43.37	
Male	220	53.14	679	56.63	
Clinical stages					
I	137	33.09	/	/	
II	116	28.02	/	/	
III	94	22.71	/	/	
IV	49	11.84	/	/	
NA	18	4.35	/	/	

SD, standard deviation; NA, not available.

[a] Two-sided χ^2 test for distributions between Wilms tumor patients and cancer-free controls.

Table S2. Potential function of the three selected SNPs in *YTHDF3* gene as predicted by SNPinfo software

rs	Chr	Allele	Location	Position	TFBS	miRNA (miRanda)	Nearby Gene	Distance (bp)	Allele	Asian	CHB
rs2241753	8	A/G	5' UTR	64243040	Y	--	LOC100128540‖YTHDF3	-29843‖-635	G	0.606	0.580
rs2241754	8	A/G	5' UTR	64243387	Y	--	LOC100128540‖YTHDF3	-30190‖-288	A	0.596	0.643
rs7464	8	A/G	3' UTR	64287510	--	Y	YTHDF3	43835‖390	A	0.774	0.747

SNP, single nucleotide polymorphism; TFBS, transcription factor binding site; CHB, Han Chinese in Beijing, China; UTR, untranslated region.

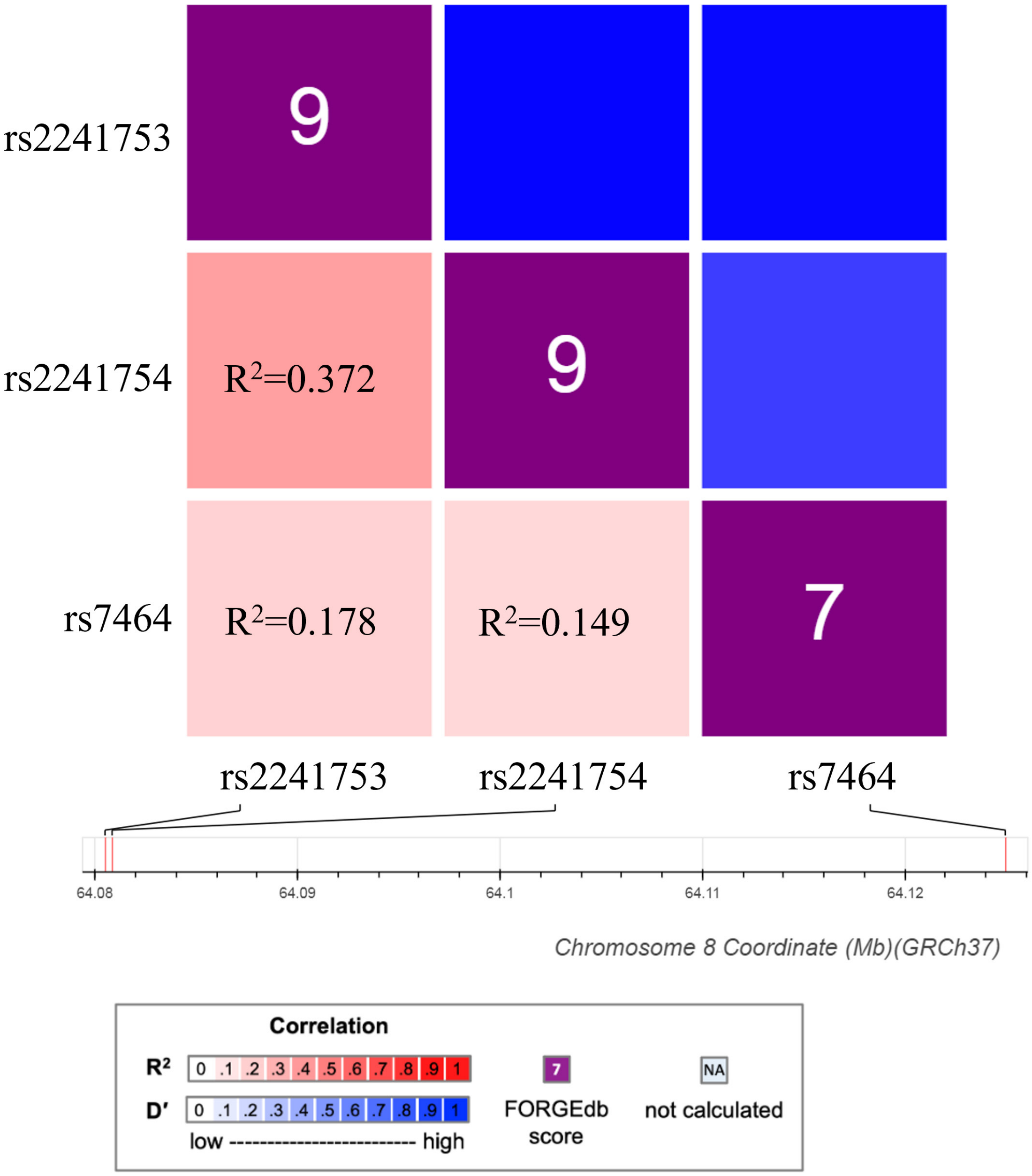

Figure S1. Linkage disequilibrium (LD) analysis for the three selected polymorphisms in Chinese Han population consisting of CHB (Han Chinese in Beijing, China) and CHS (Southern Han Chinese) subjects. LD as R^2 for polymorphism pairs is shown inside the squares.
